# Classification of Parkinson’s disease and its stages using machine learning

**DOI:** 10.1038/s41598-022-18015-z

**Published:** 2022-08-18

**Authors:** John Michael Templeton, Christian Poellabauer, Sandra Schneider

**Affiliations:** 1 Department of Computing and Information Sciences, Florida International University, Miami, FL 33199 USA; 2 Department of Communicative Sciences and Disorders, Saint Mary’s College, Notre Dame, IN 46556 USA

**Keywords:** Machine learning, Neurological disorders

## Abstract

As digital health technology becomes more pervasive, machine learning (ML) provides a robust way to analyze and interpret the myriad of collected features. The purpose of this preliminary work was to use ML classification to assess the benefits and relevance of neurocognitive features both tablet-based assessments and self-reported metrics, as they relate to Parkinson’s Disease (PD) and its stages [Hoehn and Yahr (H&Y) Stages 1–5]. Further, this work aims to compare perceived versus sensor-based neurocognitive abilities. In this study, 75 participants ($n = 50$ PD; $n = 25$ control) completed 14 tablet-based neurocognitive functional tests (e.g., motor, memory, speech, executive, and multifunction), functional movement assessments (e.g., Berg Balance Scale), and standardized health questionnaires (e.g., PDQ-39). Decision tree classification of sensor-based features allowed for the discrimination of PD from healthy controls with an accuracy of $92.6\%$, and early and advanced stages of PD with an accuracy of $73.7\%$; compared to the current gold standard tools [e.g., standardized health questionnaires ($78.3\%$ accuracy) and functional movement assessments ($70\%$ accuracy)]. Significant features were also identified using decision tree classification. Device magnitude of acceleration was significant in 12 of 14 tests ($85.7\%$), regardless of test type. For classification between diagnosed and control populations, 17 motor (e.g., device magnitude of acceleration), 9 accuracy (e.g., number of correct/incorrect interactions), and 8 timing features (e.g., time to between interactions) were significant. For classification between early (H&Y Stages 1 and 2) and advanced (H&Y Stages 3, 4, and 5) stages of PD, 7 motor, 12 accuracy, and 14 timing features were significant. Finally, this work depicts that perceived functionality of individuals with PD differed from sensor-based functionalities. In early-stage PD was shown to be $21.6\%$ lower than sensor-based scores with notable perceived deficits in memory and executive function. However, individuals in advanced stages had elevated perceptions (1.57*x*) for executive and behavioral functions compared to early-stage populations. Machine learning in digital health systems allows for a more comprehensive understanding of neurodegenerative diseases and their stages and may also depict new features that influence the ways digital health technology should be configured.

## Introduction

As the prevalence of digital health technology and the subsequent collection of large amounts of complex health data increases, machine learning (ML) methods provide epidemiologists with a robust way to analyze and interpret relevant patterns^1,2^. The increased availability of electronic health data and the use of machine learning models presents major opportunities in the healthcare space for discovery, improving patient safety, and the quality of care for individuals with neurodegenerative diseases^3–6^. Moreover, this digital health technology has become increasingly widespread in the area of neurocognitive assessments^7–9^. Mobile device capabilities allow for the collection of more objective information (e.g., digital biomarkers) than is currently achievable using pen-and-paper style tests^10,11^. In addition, digital health technology allows for the implementation of standardized health screenings and patient reported outcomes (PROs) for individual evaluation^12^. Previous work has identified that device-based sensors and/or user-device interactions used in digital assessments (e.g., accelerometry based gait assessments, speech recognition systems, and PROs for healthcare) enhances the utility and quality of this collected data^2^. Further, the combination of objective metrics and patient reported outcomes allows for the collection of relevant health information and monitoring of all functional areas of neurocognition (e.g., motor, memory, speech, language, executive function, autonomic function, sensory, behavior, and sleep)^13^. This paper focuses on individuals with Parkinson’s Disease (PD) as they may demonstrate impaired functionality across each of these functional areas of neurocognition^14^.

Mobile devices allow for the implementation of digital versions of standardized assessments (e.g., Montreal Cognitive Assessment (MoCA)^15^; Mini Mental State Examination (MMSE)^16^) and questionnaires (e.g., PDQ-39^17^) on mobile devices for the collection of these objective digital biomarkers and relevant PROs^18^. Subsequently, these mobile devices can utilize machine learning on the aforementioned feature sets for the depiction of an individual’s neurocognitive functionality, quality of life, and quantification of disease progression^13,19,20^. As the volume of relevant health data increases, novel ways to interact with and extract meaning from the data emerge^1,21^. Machine learning is a key technique that has demonstrated the ability to translate these large health data sets into actionable knowledge^4,22^. Specifically, supervised machine learning of health data has shown potential in the area of disease prediction and classification^23,24^. In supervised machine learning problems, the utilization of clinically relevant and objective features is necessary as the performance of these algorithms is heavily dependent upon the quality of the input features^25^. Therefore, the aim of these digital health systems should be to increase the reliability and accuracy of patient reported data by combining it with objective data from mobile devices, through maintaining commonly utilized methods to monitor patients’ short-term (e.g., day to day) changes in their condition and minimizing individual variability and/or bias from subjective reporting methods^18,26–29^.

The objective of this preliminary work was to use supervised machine learning classification for the assessment of novel features gathered from specifically-designed tablet-based digital neurocognitive assessments (e.g., digital biomarkers) as they relate to Parkinson’s Disease and its stages (Hoehn and Yahr Stages 1–5)^30^. In addition, commonly used self-reported metrics (e.g., from specifically designed questionnaires) and clinically-relevant functional movement assessments were included in the supervised machine learning process. Decision tree classification was used to gather significant, objective features (e.g., novel digital biomarkers) for both disease classification (e.g., whether an individual has PD) and stage classification (e.g., what stage of PD are they in). Finally, this work visualized individuals’ perceived neurocognitive capabilities (e.g., responses from subjective questionnaires) in comparison to sensor-based neurocognitive functionality scores (e.g., completion of functional assessments) between groups with and without PD to further depict the necessity of digital health systems as they relate to digitally collected health features.

## Methods

### Disease staging scales

Parkinson’s Disease rating scales are a means of assessing the symptoms of the condition by providing information on the course of the condition and/or assessment of an individual’s quality of life. Disease severity was collected in accordance with the Hoehn and Yahr Scale (H&Y)^31^ (i.e., an internationally used PD progression rating method for clinical practice) and the MDS-Unified Parkinson’s Disease Rating Scale (MDS-UPDRS)^32^ (i.e., a scale developed to incorporate elements from existing scales to provide an efficient, flexible, and comprehensive means to monitor both motor and non-motor PD symptoms)^33^.

Due to its strong clinimetric performance for motor assessments, a high correlation with MDS-UPDRS scores while minimizing intra-subject variance, in addition to providing a concise means of summarizing patient status, the H&Y staging scale was used in the classification of individuals in this preliminary work to maintain heterogeneity and efficiency^30,33,34^. The stages of the H&Y scale are listed below:

- Stage 1: Symptoms are present on one side only (unilateral).
- Stage 2: Symptoms are present on both sides but no impairment of balance.
- Stage 3: Balance impairment and mild to moderate disease progression.
- Stage 4: Severe disability, but still able to walk or stand unassisted.
- Stage 5: Needing a wheelchair or bedridden unless assisted.

### Cohort

Seventy-five adults between the ages of 50 and 85, divided into two groups- those with a confirmed diagnosis of Parkinson’s Disease and age-matched healthy controls participated in this study. The PD population included 50 individuals; with 22 being in confirmed early stages of PD (H&Y Stages 1 and 2), 9 being in confirmed advanced stages of PD (H&Y Stages 3, 4, and 5), and the remaining 11 were unaware of what stage of the condition they were in (e.g., their respective stage was not communicated to them via a licensed clinician). A breakdown of the population is shown in Table 1. Of the group of individuals diagnosed with PD, slightly more than half were female (n = 26 or 52%). The age-matched control population included 25 individuals; with slightly less than half (n = 12 or 48%) being female. Participants were recruited through advertisements, designed rehabilitation programs, physician and clinician referrals, spouses or caretakers of the diagnosed population, and prior studies from our laboratory. As the mean onset age for PD in the Western world is early-to-mid 60s^35^, recruitment efforts for this study were limited to individuals in the aforementioned groups aged 50 years or older. Participants were excluded from the current study if they were unable to provide informed consent or if they were unable to speak and/or understand English (as all instructions and tests were formatted in English). All methods in this study were performed in accordance with the relevant guidelines and regulations from the Institutional Review Board (IRB) for the protection of human subjects.

**Table 1:** Cohort breakdown.

Group	Population
**Individuals with confirmed diagnosis**	50
Individuals in early stages of PD	22
Individuals in advanced stages of PD	9
Individuals in unknown stages of PD	11
Age matched control population	25

### Qualitative assessment questionnaires

All participants were given a set of questions commonly administered in clinical settings for aging populations in addition to questions specifically asked in the event of suspected neurodegenerative disease. Commonly administered questions included how the individual felt in general, their energy levels, their pain level, their sleep quality, in addition to rating their cognitive functions of memory, speech, motor, and executive functions. During data collection, patients were also asked to give information regarding when, relative to taking medication, their data was collected. The rationale for this parameter is that the assessment of the patient near trough levels will depict the most extreme effects of the PD without the effect of any medication. Finally, a specific quality of life questionnaire (e.g., PDQ-39) was used in which the individual assesses their mobility, activities of daily living, emotional well-being, stigma, social support, cognition, communication, and bodily discomfort^17^. This standardized PDQ-39 begins each question with “Due to having Parkinson’s disease, how often during the last month have you...”. For the control group a modified version of the PDQ-39 (e.g., removing ‘Due to having Parkinson’s disease’ such that the question reads ‘How often during the last month have you...’) was given to understand quality of life over the same functional areas. In this modified version for the control population, a single question, specific only to the PD population (e.g., ‘How often during the last month have you felt you had to conceal your Parkinson’s from people?’), was removed.

### Functional movement assessments

Participants in the PD group were also administered multiple functional movement assessments both subjective and objective in nature as part of regularly scheduled clinical measures. The Berg Balance Scale, Timed Up and Go (TUG), Sit to Stand (STS), and Six Minute Walk Test (6MWT) are all commonly used functional movement assessments administered by clinicians (e.g., physical trainers or therapists) to assess functional performance^36–39^.

### Mobile application testing

All participants were administered, by clinicians (e.g., physical trainers or therapists), a tablet-based neurocognitive assessment specifically designed for individuals with Parkinson’s Disease that focused on user-device interactions for the collection of novel and objective metrics^40^. This was completed to maintain a controlled setting and ensure correct understanding of and compliance with instructions^3^. Each participant completed mobile versions of 14 neurocognitive functional tests across the areas of motor, memory, speech, and executive function. Functional tests included single functional tests (e.g., having focus on only one area of neurocognition; motor or memory) and multifunctional tests (e.g., combining two or more single functional tests into one functional test). The 14 administered neurocognitive tests collected 208 objective tablet-based digital biomarkers for all participants. All test descriptions are listed.

- For a fine-motor tracing task the individual is instructed to use their index finger to trace a depicted shape (e.g., a circle).
- In a gross-motor task the user is to manipulate the tablet to “air”-trace a prompted shape (e.g., a square).
- For reaction tasks, the user is intended to tap on the screen to interact with a set of targets.
- For a set of card matching tasks the user is to tap on depicted cards until all cards have been matched in pairs.
- For a set of speech-based tasks, the user is instructed to read a sentence and passage out loud, and name prompted objects.
- In a set of trail making tasks the user is intended to draw a line using their index finger to connect the shapes in increasing numerical order.
- A set of multifunctional tasks include a motor task (e.g., tracing or emulating an object) paired with a non-automatic speech task (e.g., listing the months of the year, aloud, in reverse order; December to January).
- For an executive function/multifunctional task a digital version of the Stroop Word Color Test (SWCT)^41^ was utilized where the user was required to discern the difference between prompted colors and words and then speak the correct response.
- In an expanded multifunctional task approach (e.g., Narration Writer), the user was instructed to narrate a sentence (e.g., speech) while also writing (e.g., motor) word by word (e.g., writing the same word being said aloud) in the space provided (e.g., executive function).

### Machine learning

The use of decision trees is considered highly powerful in classification problems and there are many popular decision tree algorithms (e.g., CART, ID3, C4.5, CHAID, and J48)^42^. For this preliminary work, the Classification and Regression Tree (CART) algorithm was chosen due to its high model interpretability, minimization of misclassification, and its diagnostic performance (e.g., increasing use in diagnosis and staging classification problems with respect to medicine, especially in situations where the underlying population is partitioned into a relatively small number of subgroups with distinct means)^43–47^. Further, this algorithm was chosen as classification and regression trees are highly common supervised methods and they are traditionally used in the selection of optimal training samples for future machine learning models^43,48–50^. This preliminary work utilized an optimized version of the CART algorithm implemented via the ‘Scikit-Learn’ Python module. The CART algorithm constructs binary trees using the feature and threshold that yields the largest information gain at each node^51^. The CART algorithm uses the Gini Index as a metric to originate binary splits. The calculation of the Gini Index is depicted in Equation (1) where $P_i$ is the probability of an object being classified to a particular class.

1 $$\begin{aligned} Gini \, Index = 1 - \sum _{i=1}^{C} (P_{i})^{2} \end{aligned}$$

The Gini Index will always be between 0 and 1 where a value of 0.5 shows an equal distribution of elements over classes, and a value closer to 0 will depict a better binary split.

### Feature normalization

Finally, the standardization of feature values is necessary to further understand the gap between self reported information and objective features as it relates to current clinical applications^13^. As graphic visualizations have enormous potential to promote patient-centered care^52^ this feature normalization was completed as part of a tandem processing step to depict differences in perceived PROs and device collected, sensor-based functionality scores (e.g., as many features are of unique type and have varying units). For this normalization, Z-scores were used. The calculation of a Z-score is depicted in Eq. (2) where x is an individual’s score, $\mu$ is the population mean and $\sigma$ is the standard deviation of the population.

2 $$\begin{aligned} Z = \dfrac{(x- \mu )}{\sigma }\end{aligned}$$

The Z-score is measured in terms of standard deviations from the mean. If a Z-score is 0, it indicates that the data point’s score is identical to the mean. A Z-score of 1.0 would indicate a value that is one standard deviation from the mean. Z-scores may be positive or negative, with a positive value indicating the score is above the mean and a negative score indicating it is below the mean.

### Ethical approval

The work presented in this manuscript is part of an approved study by The University of Notre Dame and Florida International University Institutional Review Boards (IRBs) for the Protection of Human Subjects. All methods were performed in accordance with the relevant guidelines and regulations from the IRBs. Written informed consent was collected from all participants included in this study. The collected data was authorized for disclosure as part of published works.

## Results

### Disease classification

Decision tree classification between groups (e.g., individuals with PD and controls) was completed for both perceived (e.g., self reported outcomes) and new objective assessments (e.g., digital biomarkers from specifically designed tablet-based functional assessments^40^). This classification between groups was done to gather further insights on which reported and objective features are significant in the classification of Parkinson’s Disease. Further, it was completed to give insights on an individual’s perceived versus sensor-based neurocognitive functionalities in subsequent analysis. Table 2 reports accuracy, precision, and recall for the classification between individuals with PD and control populations.

**Table 2:** Accuracy, precision, recall for classification of individuals with PD and control populations for overall assessments and subfunctional neurocognitive areas.

Assessment/function	Accuracy	Precision	Recall
**General questionnaire**	0.9024	0.925	0.9737
**PDQ-39**	1.0	1.0	1.0
**Tablet-based assessment**	0.9259	1.0	0.9130
Motor	0.8158	0.8824	0.9091
Memory	0.7632	0.8529	0.8788
Speech	0.9091	0.9474	0.9474
Executive function	0.9545	1.0	0.9474
Multifunctional	0.9630	1.0	0.9565

#### Patient reported outcomes

Patient reported outcomes (e.g., from general health questionnaires and the PDQ-39) for disease classification were analyzed in Table 2 in addition to Appendix Table 4. Decision tree classification for both the general health questionnaire and PDQ-39 depicted the most significant questions to be:

- “Do you have any handwriting problems?”
- “What is your energy level?”
- “(Due to having Parkinson’s Disease) How often in the last month have you had difficulty writing clearly?”
- “(Due to having Parkinson’s Disease) How often in the last month have you had a fear of falling?”

#### Objective digital biomarkers

Objective digital biomarker classification outcomes are presented in Table 2 as well as in Appendix Tables 5 and 6. Objective features from digital versions of 14 functional assessments were used in the classification between groups. Decision trees were generated to discern what tests are the most relevant, while also identifying what objective digital features are the most significant within each test. All accuracy, precision, and recall metrics are presented in Table 2 based on functional areas of interest (e.g., motor, memory, speech, executive function, and multifunctional assessments). Among the collected sensor-based functional tasks, multifunctional, executive functional, and speech-based tasks provided the highest accuracy, precision, and recall results in the separation of PD and control populations.

**Figure 1:**
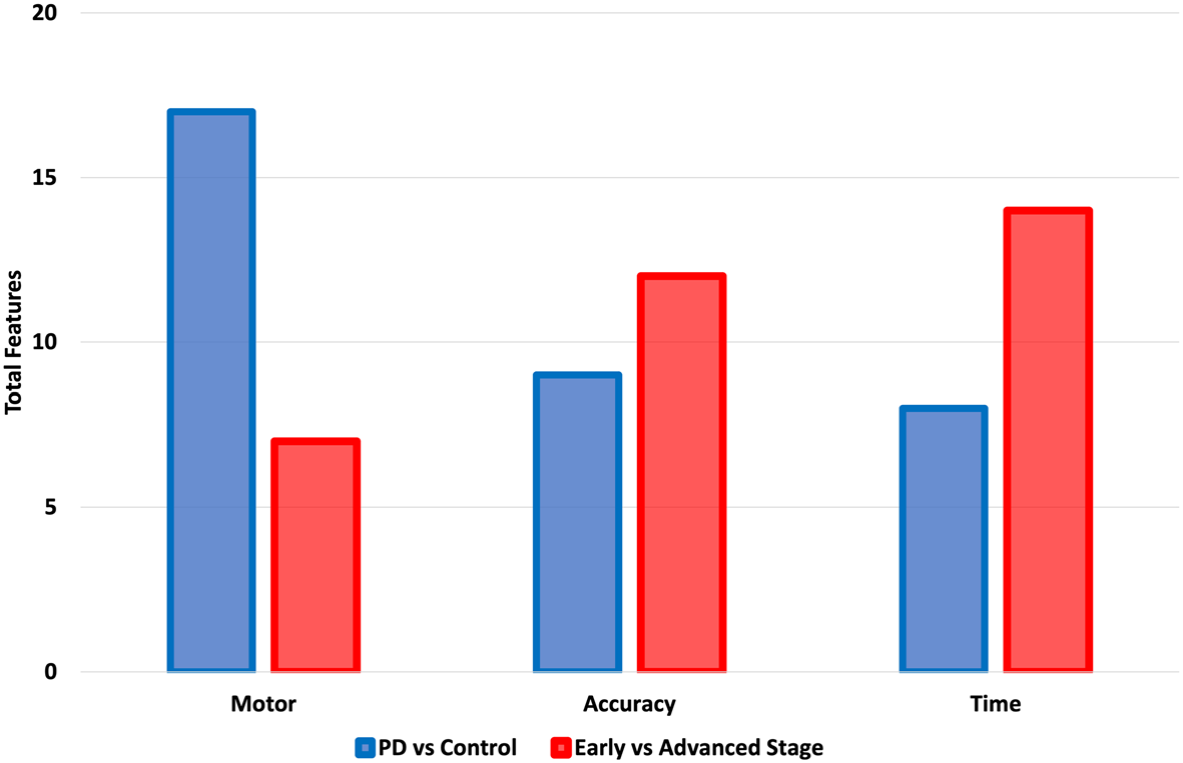
Breakdown of sensor-based scores across motor, memory, speech, executive function, and multifunctional areas of neurocognition among groups of control, early (H&Y Stages 1 and 2), and advanced stages (H&Y Stages 3, 4, and 5) of PD.

Decision tree classification also distinguished on a task level that the finger tapping test was the most significant of the administered tests in the separation of control and PD groups (Gini Index = 0.375 at the root). Other task-level assessments of importance in the separation of PD and control populations were the Grandfather Passage (Gini Index = 0.423 at the root), and multifunctional task assessments of fine motor tracing with speech (Gini Index = 0.429 at the roots). Finger tapping and multifunctional task assessments of fine motor tracing with speech had root features of device acceleration (e.g., the magnitude of acceleration of the device or how the user moves the device during the test) whereas the Grandfather Passage had an accuracy feature (e.g., the number of missed words in a speech test) as the root. In an expansion to include first order features, 17 motor, 9 accuracy, and 8 timing features are shown to be significant for the the classification between individuals diagnosed with PD and control populations. This is seen in Fig. 1.

### Stage classification

The classification of disease stage (e.g., early (H&Y Stages 1 and 2) versus advanced-stage (H&Y Stages 3, 4, and 5) Parkinson’s Disease was completed across perceived (e.g., PROs) and objective assessments (e.g., sensor-based digital biomarkers), as well as clinically administered functional assessments (e.g., Berg Balance Scale, STS, TUG, and 6MWT) for all individuals with PD. Table 3 reports accuracy, precision, and recall for the classification between individuals in early and advanced stages of PD.

**Table 3:** Accuracy, precision, recall for classification of individuals with PD in early and advanced stages of PD for overall assessments and subfunctional neurocognitive areas.

Assessment/function	Accuracy	Precision	Recall
**General questionnaire**	0.6956	0.7647	0.8125
**PDQ-39**	0.7826	0.8666	0.8125
**Functional movement assessment**	0.7	0.75	0.8571
**Tablet-based assessment**	0.7368	0.8	0.8571
Motor	0.8788	0.9524	0.8696
Memory	0.6842	0.7857	0.7857
Speech	0.8947	0.9286	0.9286
Executive function	0.7368	0.8461	0.7857
Multifunctional	0.8421	0.9231	0.8571

#### Patient reported outcomes and functional assessments

Patient reported outcomes and clinically administered functional assessment results for stage classification are seen in Table 3 as well as in Appendix Table 7. PROs come from general health questionnaires and the PDQ-39, whereas functional assessment results come from the Berg Balance Scale, TUG, STS, and 6MWT. Decision tree classification for the PROs depicted the most significant questions in the classification of PD stage to be:

- “Do you have any handwriting problems?”
- “What is your energy level?”
- “Due to having Parkinson’s Disease how often in the last month have you had difficulty with leisure activities?”
- “Due to having Parkinson’s Disease how often in the last month have you felt unable to communicate with others?”
- “Due to having Parkinson’s Disease how often in the last month have you felt unpleasantly hot or cold?”

Classification of functional assessment features from the Berg Balance Scale depicted the most significant features to be standing with one foot in front and turning to look behind, whereas objective functional movement features included the distance traveled during the 6 Minute Walk Test and the individual’s speed during the Timed Up and Go.

#### Objective digital biomarkers

Objective digital biomarker outcomes for stage classification can be seen in Table 3 in addition to Appendix Tables 8 and 9. Similar to disease classification, the breakdown between stages (e.g., early and advanced-stage Parkinson’s Disease) was completed using collected objective features from digital versions of functional assessments. All accuracy, precision, and recall metrics are presented in Table 3 based on functional areas of interest (e.g., motor, memory, speech, executive function, and multifunctional assessments). Among the collected sensor-based functional tasks, speech, motor, and multifunctional tasks provided the highest accuracy, precision, and recall results in the separation of early and advanced stage PD populations.

Decision tree classification also distinguished on a task level that 11 of 14 administered tests were significant in the separation of early (H&Y Stages 1 and 2) and advanced stages (H&Y Stages 3, 4, and 5) of Parkinson’s Disease (Gini Index = 0.363 at the root). Tests with Gini Index higher than 0.363 include Trail Making Tests (e.g., executive function) and the Grandfather Passage with Gini Index values of 0.375 and 0.423, respectively. Root features from the 11 significant tests included 1 device acceleration feature (e.g., how the user moves the device during the test), 4 timing features (e.g., the total elapsed speaking time, or average time between non-match pair), and 6 accuracy features (e.g., the number of targets tapped, or total correct objects named). In an expansion to include first order features, 7 motor, 12 accuracy, and 14 timing features best separate groups based on stage; the inverse of diagnosis. This is also seen in Fig. 1.

### Feature normalization

As graphic visualizations have enormous potential to promote patient-centered care^52^ feature normalization was completed as part of a tandem processing step to depict differences in perceived PROs and device collected, sensor-based functionality scores. This standardization is necessary to further understand the gap between self reported information and objective features as it relates to current clinical applications^13^ and support the preface that the aim of digital health systems should be used to increase the reliability and accuracy of patient reported data by combining it with objective data from mobile devices^18,26–29^.

#### Patient reported outcomes

**Figure 2:**
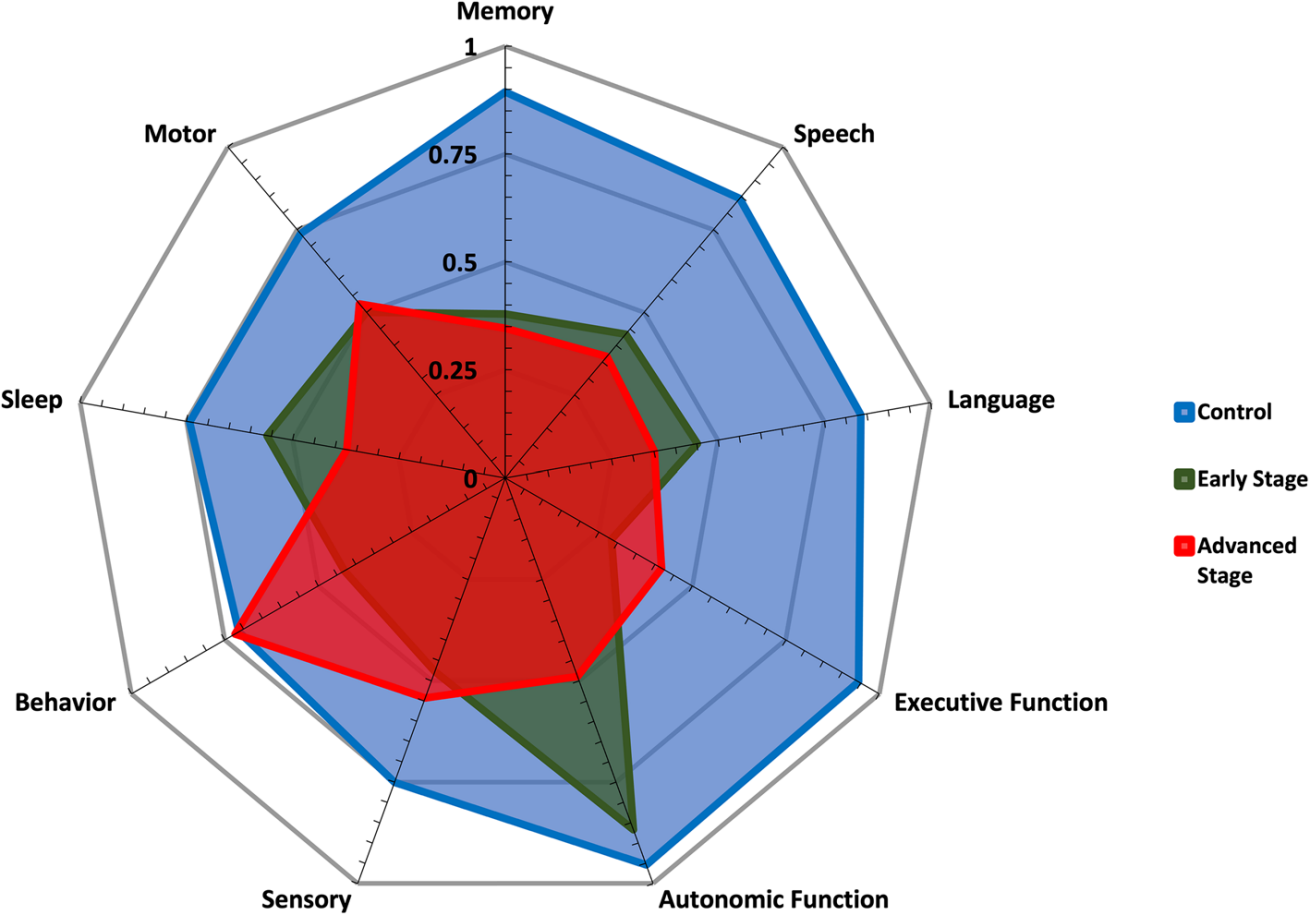
Breakdown of perceived functionality across all functional areas of neurocognition among groups of control, early (H&Y Stages 1 and 2), and advanced stages (H&Y Stages 3, 4, and 5) of PD.

In the depiction of perceived neurocognitive functionalities between groups, normalized scores from general health questionnaires and the PDQ-39 were calculated for all functional areas of neurocognition. Z-scores were used in the standardization of these features. This standardization of feature values is necessary as many features are of a unique type and have varying units. The weighted Z-scores of perceived capabilities of controls, early-stage PD (H&Y Stages 1 and 2), and advanced-stage PD (H&Y Stages 3, 4, and 5) populations are shown in Fig. 2.

#### Objective digital biomarkers

**Figure 3:**
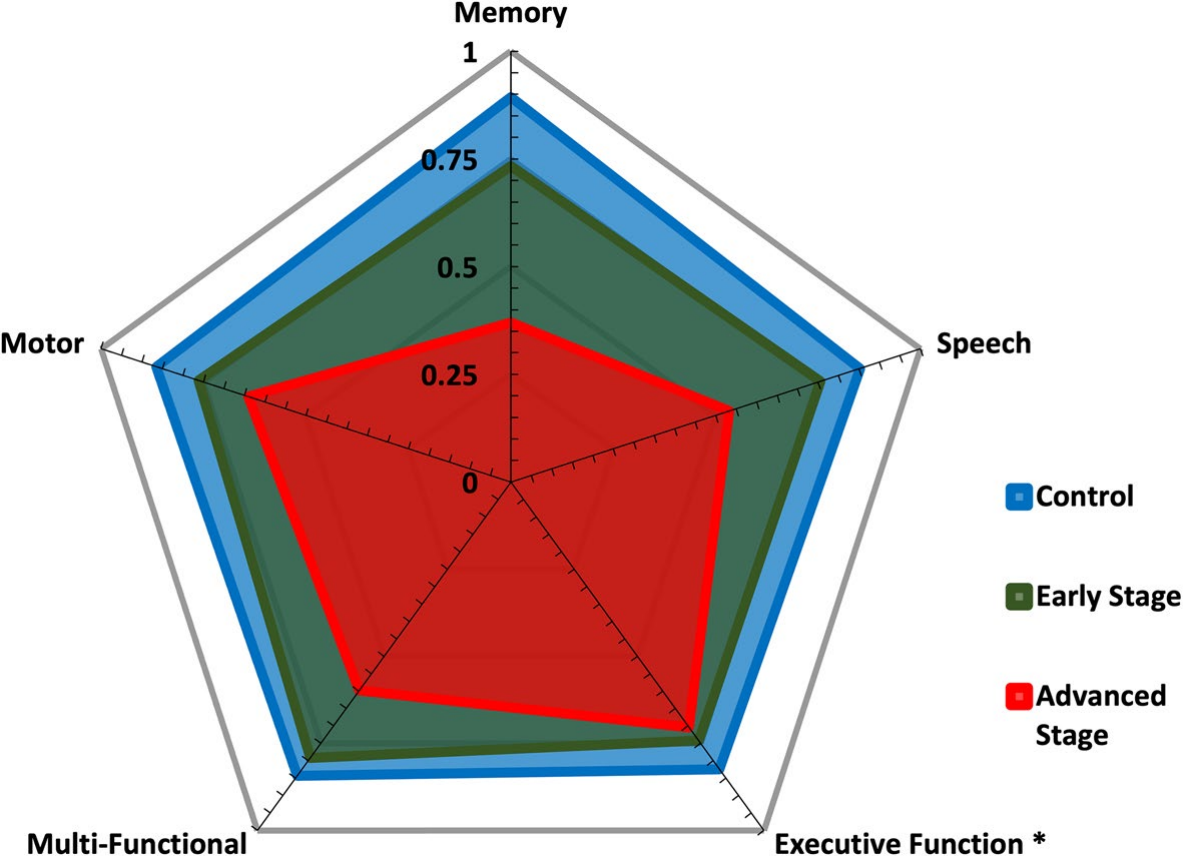
Breakdown of sensor-based functionality scores across motor, memory, speech, executive function, and multifunctional areas of neurocognition among groups of control, early (H&Y Stages 1 and 2), and advanced stages (H&Y Stages 3, 4, and 5) of PD.

For sensor-based neurocognitive functionalities between groups, normalized scores from objective assessments were calculated for the functional areas of motor, memory, speech, executive function, and multifunctional tests. The weighted Z-scores of sensor-based neurocognitive capabilities for controls, early-stage PD (H&Y Stages 1 and 2), and advanced-stage PD (H&Y Stages 3, 4, and 5) populations are shown in Fig. 3. It should be noted that all executive function tests are inherently multifunctional in nature (e.g., an individual needs to move or speak to carry out the executive function) and therefore are a subset of the multifunctional test digital biomarker set (e.g., denoted by an * in Fig. 3).

## Discussion

As Parkinson’s Disease is often described as a “designer disease”, meaning individuals with PD manifest different symptoms across the spectrum of disease characteristics, personalized medicine should be the goal and is required to optimize care^53,54^. However, to reach personalized medicine utilizing machine learning, relevant features need to be identified as the performance of given algorithms are heavily dependent upon the quantitative and quality of the extracted features^4^. Nearly 275 features were collected in this work from PROs (e.g., from the PDQ-39), functional movement assessments (e.g., from the TUG, STS, and 6MWT), and novel objective digital biomarkers (e.g., from tablet-based assessments) across multiple neurocognitive tasks. This work sought to identify new significant features in the classification of individuals with Parkinson’s Disease compared to controls (e.g., what features are the most important in discerning if an individual has PD or not), as well as the classification of different stages of PD (e.g., what features best aid the depiction of how far the disease has progressed).

Commonly, PROs are used to monitor an individual’s thoughts or opinions on changes in their condition which can lead to improved disease management in the recognition and understanding of their symptoms and triggers^18,26^. However, this perceived information may be subject to individual variability and/or bias^27–29^. This work depicts variability in perceived functionality scores (e.g., motor, memory, speech, and executive function) from sensor-based scores for some groups. The perceived functionality for individuals in confirmed early-stage PD (H&Y Stages 1 and 2) across the areas of memory, speech, and executive function, differs by about 22% compared to their sensor-based functionality scores as shown in Figs. 2 and 3. Further, these figures show a relatively large perceived increase in executive function and behavioral abilities for individuals in advanced stages of PD (H&Y Stages 3, 4, and 5) compared to their early-stage counterparts. Therefore these digital health systems, with the ability to administer, collect, and subsequently analyze objective features, should be utilized in a way to allow individuals greater insights on their true capabilities.

Decision tree classification brought to light new metrics that expand the way digital versions of functional assessments should be configured. This is first discussed at the most granular level (e.g., task specific). Although primary manifestations of PD include abnormalities of movement (e.g., akinesia, rigidity, and tremor), pen-and-paper style assessments cannot measure all of these abnormalities in the same manner as digital technology^10,13,55^. The way the individual moves the device during assessments (e.g., voluntarily and/or involuntarily) proves to be a significant feature in the classification between individuals with PD and control populations. Device magnitude of acceleration (e.g., maximum, minimum, or average magnitude of acceleration) was a significant (e.g., root or first order) feature in 12 of 14 tests (85.7%), regardless of test type (e.g., motor, memory, speech, executive function or multifunctional tests). Magnitude of acceleration features are unable to be collected in pen-and-paper style assessments, nor are they currently being collected across all functional tests in implemented digital versions of clinical assessments. Conversely, variably present manifestations (e.g., dysarthria and difficulty performing simultaneous actions^55^) are able to be seen in current pen-and-paper versions or other clinically implemented assessments. These difficulties can be depicted in timing and accuracy features. However, these depictions are more significant in the delineation between stages (e.g., early versus advanced-stage PD). Features of timing (10 of 14 tests; 71.43%) and accuracy (8 of 14 tests; 57.14%) are significant (e.g., root or first order) features in classification of stage, whereas only 5 of 14 tests (35.71%) contain significant device motion features. Mobile-based versions of neurocognitive assessments have the capability to expand clinically relevant functional assessments^56,57^. Employing opportunistic approaches to monitoring (e.g., having device sensors on in the background regardless of test type) allows for the collection of novel objective features^13,58^. An example shown in this work was the use of the device accelerometer across all functional tests. However, this premise could also include other device sensors such as microphones and cameras. On a functional level (e.g., across functional areas of motor, memory, speech, executive function, and multifunctional tasks); speech, executive function, and multifunctional tasks were shown to have a high accuracy in the the classification of PD compared to control populations. Further, in addition to motor tasks, speech and multifunctional tasks were found to have high accuracy for classification of differing stages of PD [e.g., early stages (H&Y Stages 1 and 2) versus advanced stages (H&Y Stages 3, 4, and 5)]. This also advocates that although primary manifestations of PD include abnormalities of movement ^10,13,55^, additional functional areas of neurocognition need to be included/expanded in assessing PD to yield a broader understanding of the disease and assist with the treatment of “designer” symptoms.

Given this preliminary work, limitations and future work are also addressed in kind. A main limitation of this work is that some study participants were unaware of the stage of PD they were in (e.g., their respective stage was not communicated to them via a licensed clinician). This may be due to both the nature of the disease (e.g., being a “designer disease”) as well as the subjectivity in the staging criteria^30,53^. Further, given the population size, stage classification had to occur on the basis of grouped stages of Parkinson’s Disease [e.g., early (H&Y Stages 1 and 2) or advanced (H&Y Stages 3, 4, and 5)], rather than individual observed stages 1–5. Increased communication between clinicians and diagnosed populations on the advancement and severity of the disease is imperative for both rehabilitative efforts and disease monitoring. Since this preliminary work was (1) highly limited in sample size (only 56 labeled samples; 22 being confirmed early stages of PD (H&Y Stages 1 and 2), 9 being confirmed advanced stages of PD, and 25 being confirmed age-matched healthy controls), and (2) focused on aiding clinician diagnoses rather than achieving state-of-the-art performance, train-test evaluation metrics should be recalculated following the collection of much larger sample sizes for the provision of revised digital health system classification models. Following the expansion the collected dataset, extensions of this work should also include the use of additional machine learning methods (e.g., k-nearest neighbors, decision trees, logistic regression, and random forests)^20,59,60^ for classification of the disease and its stages. Another limitation of the work completed in this manuscript only shows a snapshot of Parkinson’s Disease and subsequent neurocognitive functionalities. Future work should include additional clinical assessments for the collection of further device-derived features. Future work should also analyze how different interventions (e.g., medical, pharmacological, dietary, physical, speech, and occupational therapies) affect significant features and overall functionality in comparison to controls^61^. Given the collection of additional user data with respect to intervention protocols, ML should also be used in the automation of intervention recommendations. Finally, as this preliminary work includes novel digital features that were collected and analyzed via the supervised learning model, validation against other datasets was not possible.

## Conclusions

Using digital health systems for monitoring individuals with neurodegenerative diseases allows for more comprehensive insights of these conditions and their progression. As digital health technology allows for the collection of large amounts of complex health data, ML provides a more efficient way to analyze and interpret patterns in the data. In addition, classification methods may also bring to light new features that expand the way digital versions of functional assessments should be administered. Utilizing this information to reevaluate and update standardized techniques and scales can truly allow for novel digital health systems and disease monitoring which may be helpful in the classification of individuals with different neurodegenerative conditions (e.g., Alzheimer’s Disease, ALS, multiple sclerosis, Huntington’s Disease, and other types of dementia) and their respective stages. In turn, this can aid clinicians, diagnosed populations, and caretakers in monitoring all neurocognitive functions while also allowing for increased efficacy for both diagnostic and rehabilitative purposes.

## Supplementary information

Supplementary Information.

## Data Availability

The datasets generated and/or analysed during the current study are not publicly available due to this data being part of a larger dataset for concurrent projects on mobile based neurocognitive assessments. However, the data can be made available from the corresponding author upon reasonable request.

## References

[1] Wiens J, Shenoy ES (2018). Machine learning for healthcare: On the verge of a major shift in healthcare epidemiology. Clin. Infect. Dis..

[2] Templeton, J. M., Poellabauer, C. & Schneider, S. Design of a neurocognitive digital health system (NDHS) for neurodegenerative diseases. in *Proceedings of the 2021 Workshop on Future of Digital Biomarkers* 26–33, 10.1145/3469266.3471157 (2021).

[3] Far MS, Eickhoff SB, Goni M, Dukart J (2021). Exploring test-retest reliability and longitudinal stability of digital biomarkers for Parkinson disease in the m-power data set: Cohort study. J. Med. Internet Res..

[4] Waring J, Lindvall C, Umeton R (2020). Automated machine learning: Review of the state-of-the-art and opportunities for healthcare. Artif. Intell. Med..

[5] Bates DW, Saria S, Ohno-Machado L, Shah A, Escobar G (2017). Big data in health care: Using analytics to identify and manage high-risk and high-cost patients. Health.

[6] Marella WM, Sparnon E, Finley E (2017). Screening electronic health record-related patient safety reports using machine learning. J. Patient Saf..

[7] Deng K (2022). Heterogeneous digital biomarker integration out-performs patient self-reports in predicting Parkinson’s disease. Commun. Biol..

[8] Maetzler W, Pilotto A (2021). Digital assessment at home: mPower against Parkinson disease. Nat. Rev. Neurol..

[9] Hansen C, Sanchez-Ferro A, Maetzler W (2018). How mobile health technology and electronic health records will change care of patients with Parkinson’s disease. J. Parkinson Dis..

[10] Byrom B, Wenzel K, Pierce J, Wenzel K, Pierce J (2016). Computerised clinical assessments: Derived complex clinical endpoints from patient self-report data. EPro.

[11] Templeton JM, Poellabauer C, Schneider S (2021). The Case for Symptom-Specific Neurological Digital Biomarkers.

[12] Kumar S (2013). Mobile health technology evaluation. Am. J. Prev. Med..

[13] Templeton JM, Poellabauer C, Schneider S (2020). Enhancement of neurocognitive assessments using smartphone capabilities: Systematic review. JMIR mHealth and uHealth.

[14] Löfgren N, Conradsson D, Rennie L, Moe-Nilssen R, Franzén E (2019). The effects of integrated single- and dual-task training on automaticity and attention allocation in Parkinson’s disease: A secondary analysis from a randomized trial. Neuropsychology.

[15] Nasreddine ZS (2005). The montreal cognitive assessment, MoCA: A brief screening tool for mild cognitive impairment. J. Am. Geriatr. Soc..

[16] Tombaugh TN, McIntyre NJ (1992). The mini-mental state examination: A comprehensive review. J. Am. Geriatr. Soc..

[17] Neff C, Wang MC, Martel H (2018). Using the PDQ-39 in routine care for Parkinson’s disease. Parkinson. Relat. Disord..

[18] Deshpande P, Sudeepthi B, Rajan S, Abdul Nazir C (2011). Patient-reported outcomes: A new era in clinical research. Perspect. Clin. Res..

[19] Zhan A (2018). Using smartphones and machine learning to quantify Parkinson disease severity the mobile Parkinson disease score. JAMA Neurol..

[20] De Vos M, Prince J, Buchanan T, FitzGerald JJ, Antoniades CA (2020). Discriminating progressive supranuclear palsy from Parkinson’s disease using wearable technology and machine learning. Gait Posture.

[21] Bhardwaj R, Nambiar AR, Dutta D (2017). A study of machine learning in healthcare. Proc. Int. Comput. Softw. Appl. Conf..

[22] Hadirah N, Anwar K, Saian R, Abu Bakar S (2021). An enhanced ant colony optimization with gini index for predicting type 2 diabetes. AIP Proc..

[23] Uddin S, Khan A, Hossain ME, Moni MA (2019). Comparing different supervised machine learning algorithms for disease prediction. BMC Med. Inform. Decis. Mak..

[24] Ricciardi C (2019). Classifying different stages of Parkinson’s disease through random forests. IFMBE Proc..

[25] Domingos P (2012). Tapping into the folk knowledge needed to advance machine learning applications. Review.

[26] Vega J (2018). Back to analogue: Self-reporting for Parkinson’s disease. J. Parkinson Dis..

[27] Nicolson PJ, Hinman RS, Wrigley TV, Stratford PW, Bennell KL (2018). Self-reported home exercise adherence: A validity and reliability study using concealed accelerometers. J. Orthop. Sports Phys. Ther..

[28] Reychav I (2019). How reliable are self-assessments using mobile technology in healthcare? The effects of technology identity and self-efficacy. Comput. Hum. Behav..

[29] Prince SA (2020). A comparison of self-reported and device measured sedentary behaviour in adults: A systematic review and meta-analysis. AIP Proc..

[30] Hoehn MM, Yahr MD (1967). Parkinsonism. Neurology.

[31] Padman N, Swarnalatha R, Venkatesh V, Kumar N (2020). Telediagnosis of Parkinson’s disease symptom severity using H&Y scale. J. Eng. Sci. Technol..

[32] Goetz CG (2008). Movement disorder society-sponsored revision of the unified Parkinson’s disease rating scale (MDS-UPDRS): Scale presentation and clinimetric testing results. Mov. Disord..

[33] Martinez-Martin P (2018). Validation study of the hoehn and yahr scale included in the MDS-UPDRS. Mov. Disord..

[34] Evers LJ, Krijthe JH, Meinders MJ, Bloem BR, Heskes TM (2019). Measuring Parkinson’s disease over time: The real-world within-subject reliability of the MDS-UPDRS. Mov. Disord..

[35] Post B (2020). Young onset Parkinson’s disease: A modern and tailored approach. J. Parkinson Dis..

[36] Qutubuddin AA (2005). Validating the Berg Balance Scale for patients with Parkinson’s disease: A key to rehabilitation evaluation. Arch. Phys. Med. Rehabil..

[37] Bhatt T, Yang F, Mak MK, Hui-Chan CW-Y, Pai Y-C (2013). Effect of externally cued training on dynamic stability control during the sit-to-stand task in people with Parkinson disease. Phys. Ther..

[38] Brusse KJ, Zimdars S, Zalewski KR, Steffen TM (2005). Testing functional performance in people With Parkinson disease. Phys. Ther..

[39] Duncan RP, Leddy AL, Earhart GM (2011). Five times sit to stand test performance in Parkinson disease. Arch. Phys. Med. Rehabil..

[40] Templeton, J. M., Poellabauer, C. & Schneider, S. *Design of a Mobile-Based Neurological Assessment Tool for Aging Populations*. 166–185, (Springer, 2021). 10.1007/978-3-030-70569-5_11.

[41] Scarpina F, Tagini S (2017). The stroop color and word test. Front. Psychol..

[42] Dangare CS, Apte SS, Student ME (2012). Improved study of heart disease prediction system using data mining classification techniques. Int. J. Comput. Appl..

[43] Aydın F, Aslan Z (2021). Recognizing Parkinson’s disease gait patterns by vibes algorithm and Hilbert–Huang transform. Eng. Sci. Technol. Int. J..

[44] de Andrade JBC (2021). Oxfordshire community stroke project classification: A proposed automated algorithm. Eur. Stroke J..

[45] Ghiasi MM, Zendehboudi S, Mohsenipour AA (2020). Decision tree-based diagnosis of coronary artery disease: CART model. Comput. Methods Prog. Biomed..

[46] Venkatasubramaniam A (2017). Decision trees in epidemiological research. Emerg. Themes Epidemiol..

[47] Sharma A, Scholar R, Professor A, Gupta M (2016). Theoretical study of decision tree algorithms to identify pivotal factors for performance improvement: A review theoretical study of decision tree algorithms to identify pivotal factors for performance improvement: A review Pooja Gulati. Int. J. Comput. Appl..

[48] Kino S (2021). A scoping review on the use of machine learning in research on social determinants of health: Trends and research prospects. SSM Popul. Health.

[49] Sharma SR, Singh B, Kaur M (2021). Classification of Parkinson disease using binary Rao optimization algorithms. Expert Syst..

[50] Li Y (2017). Envelope learning view project intelligent algorithm and system view project classification of Parkinson’s disease by decision tree based instance selection and ensemble learning algorithms. J. Med. Imaging Health Inform..

[51] Gordon, L. *Using Classification and Regression Trees (CART) in SAS® Enterprise Miner TM For Applications in Public Health*. (2013).

[52] Albers EA (2022). Visualization formats of patient-reported outcome measures in clinical practice: A systematic review about preferences and interpretation accuracy. J. Patient-Rep. Outcomes.

[53] Blake-Krebs, B. *When Parkinson’s Strikes Early: Voices, Choices, Resources and Treatment*, 1st ed. (HunterHouse, 2001).

[54] Ryu J, Vero J, Dobkin RD, Torres EB (2019). Dynamic digital biomarkers of motor and cognitive function in Parkinson’s disease. J. Vis. Exp..

[55] Mazzoni P, Shabbott B, Cortés JC (2012). Motor control abnormalities in Parkinson’s disease. Cold Spring Harbor Perspect. Med..

[56] Vianello A, Chittaro L, Burigat S, Budai R (2017). MotorBrain: A mobile app for the assessment of users’ motor performance in neurology. Comput. Methods Prog. Biomed..

[57] Maguire Á, Martin J, Jarke H, Ruggeri K (2018). Getting closer? Differences remain in neuropsychological assessments converted to mobile devices. Psychol. Serv..

[58] Jacobson NC, Weingarden H, Wilhelm S (2019). Digital biomarkers of mood disorders and symptom change. NPJ Dig. Med..

[59] Pahuja G, Nagabhushan TN (2021). A comparative study of existing machine learning approaches for Parkinson’s disease detection. IETE J. Res..

[60] Dijkhuis TB, Blaauw FJ, van Ittersum MW, Velthuijsen H, Aiello M (2018). Personalized physical activity coaching: A machine learning approach. Sensors..

[61] Templeton JM, Poellabauer C, Schneider S (2022). Towards symptom-specific intervention recommendation systems. J. Parkinson’s Dis..

