# Supplementary material for: Classification of Parkinson’s disease and its stages using machine learning
Source: Sci Rep. 2022 Aug 18;12:14036. doi: 10.1038/s41598-022-18015-z (PMC9388671; doi:10.1038/s41598-022-18015-z)
Supplement: Supplementary file 1 — Supplementary Information. [file 41598_2022_18015_MOESM1_ESM.pdf]

Appendix

**Assessment / Questionnaire**	***Feature***	***Value***	***Gini***
General Questionnaire	Root Feature		
	Handwriting Problems	x $\leq$ 0.5	0.404
	First Order Feature(s)		
	Energy Level	x $\leq$ 8.5	0.492
PDQ - 39	Root Feature		
	Difficulty Writing Clearly	x $\leq$ 1.5	0.404
	First Order Feature(s)		
	Fear of Falling	x $\leq$ 1.5	0.059

Appendix Table 1. Features of Interest given Decision Tree Results for Patient Reported Outcomes - Gini Index Values of Questionnaire Responses for PD Population versus Controls.

**Assessment**	***Feature***	***Value***	***Gini***
Finger Tapping (Fine Motor)	Root Feature		
	Minimum Magnitude of Acceleration	x $\leq$ 0.871	0.375
	First Order Feature(s)		
	Total Small Circles Tapped	x $\leq$ 35.5	0.208
Grandfather Passage (Speech)	Root Feature		
	Number of Missed Words	x $\leq$ 22.5	0.423
	First Order Feature(s)		
	Maximum Magnitude of Acceleration	x $\leq$ 1.001	0.165
	Number of Additional Words	x $\leq$ 17.5	0.202
Circle Tracing (Fine-Motor)	Root Feature		
	Maximum Drawing Speed	x $\leq$ 388.85	0.435
	First Order Feature(s)		
	Minimum Magnitude of Acceleration	x $\leq$ 0.998	0.32
Square Tracing (Fine-Motor)	Root Feature		
	Maximum Drawing Speed	x $\leq$ 454.433	0.44
	First Order Feature(s)		
	Minimum Magnitude of Acceleration	x $\leq$ 0.997	0.387
Narration (Speech)	Root Feature		
	Phrase Said Incorrectly	x $\leq$ 0.5	0.44
	First Order Feature(s)		
	Time from Start to First Word Said	x $\leq$ 2.569	0.251
	Avg Time between Words Said	x $\leq$ 0.324	0.278
Object Naming (Speech)	Root Feature		
	Total Incorrect	x $\leq$ 2.5	0.44
	First Order Feature(s)		
	Minimum Magnitude of Acceleration	x $\leq$ 1.003	0.08
	Maximum Magnitude of Acceleration	x $\leq$ 1.102	0.463
Card Matching (Memory)	Root Feature		
	Minimum Magnitude of Acceleration	x $\leq$ 0.953	0.44
	First Order Feature(s)		
	Maximum Magnitude of Acceleration	x $\leq$ 1.243	0.301
Card Matching with Cognitive Interference (Memory)	Root Feature		
	Maximum Magnitude of Acceleration	x $\leq$ 1.135	0.44
	First Order Feature(s)		
	Maximum Magnitude of Acceleration	x $\leq$ 1.048	0.434

Appendix Table 2. Features of Interest given Decision Tree Results for Single Functional Tests - Gini Index Values of Functional Neurocognitive Assessment Tests for PD Population versus Controls.

**Assessment**	***Feature***	***Value***	***Gini***
Circle Tracing with Speech (Mulit-Test)	Root Feature		
	Maximum Magnitude of Acceleration	x $\leq$ 1.079	0.429
	First Order Feature(s)		
	Minimum Magnitude of Acceleration	x $\leq$ 0.924	0.444
	Total Distance Drawn	x $\leq$ 25268.62	0.137
Square Tracing with Speech (Mulit-Test)	Root Feature		
	Maximum Magnitude of Acceleration	x $\leq$ 1.074	0.429
	First Order Feature(s)		
	Maximum Time between Words Said	x $\leq$ 2.897	0.444
	Number of Outline Crossings	x $\leq$ 19.0	0.137
Narration Writer (Mulit-Test)	Root Feature		
	Minimum Magnitude of Acceleration	x $\leq$ 0.919	0.44
	First Order Feature(s)		
	Average Magnitude of Acceleration	x $\leq$ 1.003	0.313
	Total Elapsed Writing Time	x $\leq$ 27.251	0.32
Stroop Word Color (Executive Function)	Root Feature		
	Minimum Magnitude of Acceleration	x $\leq$ 1.002	0.44
	First Order Feature(s)		
	Total Colors Generated	x $\leq$ 8.5	0.369
Visuospatial (Executive Function)	Root Feature		
	Average Drawing Speed	x $\leq$ 218.442	0.458
	First Order Feature(s)		
	Minimum Magnitude of Acceleration	x $\leq$ 1.002	0.305
	Time	x $\leq$ 8.456	0.355
Visuospatial with Cognitive Interference (Executive Function)	Root Feature		
	Average Drawing Speed	x $\leq$ 186.201	0.458
	First Order Feature(s)		
	Average Distance to Correct Point	x $\leq$ 10.351	0.342
	Maximum Time Between Objects	x $\leq$ 1.933	0.426

Appendix Table 3. Features of Interest given Decision Tree Results for Multifunctional Tests - Gini Index Values of Functional Neurocognitive Assessment Tests for PD Population versus Controls.

**Assessment / Questionnaire**	***Feature***	***Value***	***Gini***
General Questionnaire	Root Feature		
	Handwriting Problems	x $\leq$ 0.5	0.404
	First Order Feature(s)		
	Energy Level	x $\leq$ 8.5	0.492
PDQ - 39	Root Feature		
	Difficulty with Leisure Activities	x $\leq$ 2.5	0.412
	First Order Feature(s)		
	Felt Unable to Communicate	x $\leq$ 0.5	0.219
	Felt Unpleasantly Hot or Cold	x $\leq$ 0.5	0.245
Berg Balance Scale	Root Feature		
	Standing With One Foot In Front	x $\leq$ 2.5	0.412
	First Order Feature(s)		
	Turning to Look Behind	x $\leq$ 3.5	0.413
Functional Movement	Root Feature		
Assessments	*Six Minute Walk*	x $\leq$ 494.5	0.412
	First Order Feature(s)		
	Timed Up and Go	x $\leq$ 9.305	0.397

Appendix Table 4. Features of Interest given Decision Tree Results of Patient Reported Outcomes/Subjective Assessment - Gini Index Values of Functional Assessments and Questionnaire Responses for PD Populations in Early Stages (H&Y Stages 1 and 2) versus Advanced Stages (H&Y Stages 3, 4, and 5).

**Assessment**	***Feature***	***Value***	***Gini***
Finger Tapping (Fine Motor)	Root Feature		
	Total Targets Tapped	$x \leq 32.5$	0.363
	First Order Feature(s)		
	Maximum Magnitude of Acceleration	$x \leq 1.1937$	0.198
Circle Tracing (Fine-Motor)	Root Feature		
	First and Last Point Distance	$x \leq 74.77$	0.363
	First Order Feature(s)		
	Average Drawing Speed	$x \leq 251.92$	0.208
	First and Last Point Distance	$x \leq 182.21$	0.375
Square Tracing (Fine-Motor)	Root Feature		
	Minimum Magnitude of Acceleration	$x \leq 0.907$	0.363
	First Order Feature(s)		
	Maximum Drawing Speed	$x \leq 1327.25$	0.496
Narration (Speech)	Root Feature		
	Total Elapsed Speaking Time	$x \leq 5.948$	0.363
	First Order Feature(s)		
	Time from Start to First Word Said	$x \leq 3.689$	0.5
Object Naming (Speech)	Root Feature		
	Total Correct Objects	$x \leq 10.5$	0.363
	First Order Feature(s)		
	Total Incorrect Objects	$x \leq 1.5$	0.124
	Average Time for Correct Response	$x \leq 2.52$	0.444
Card Matching (Memory)	Root Feature		
	Average Time Between Non-Match Pair	$x \leq 1.98$	0.363
	First Order Feature(s)		
	Maximum Magnitude of Acceleration	$x \leq 1.372$	0.198
Card Matching with Cognitive Interference (Memory)	Root Feature		
	Average Time Between Non-Match Pair	$x \leq 2.377$	0.363
	First Order Feature(s)		
	Average Time Between Non-Match Pair	$x \leq 0.949$	0.198
Grandfather Passage (Speech)	Root Feature		
	Maximum Magnitude of Acceleration	$x \leq 1.325$	0.423
	First Order Feature(s)		
	Average Time between Words Said	$x \leq 0.584$	0.32

Appendix Table 5. Features of Interest given Decision Tree Results for Single Functional Tests - Gini Index Values of Functional Neurocognitive Assessment Tests for PD Population versus Controls.

Assessment	*Feature*	*Value*	*Gini*
Circle Tracing	Root Feature		
with Speech	*Number of Missing Months*	x $\leq$ 2.5	0.363
(Mulit-Test)	First Order Feature(s)		
	Average Distance from Shape	x $\leq$ 8.465	0.408
Square Tracing	Root Feature		
with Speech	*Number of Missing Months*	x $\leq$ 2.5	0.363
(Mulit-Test)	First Order Feature(s)		
	Maximum Magnitude of Acceleration	x $\leq$ 1.104	0.469
Narration Writer	Root Feature		
(Mulit-Test)	*Average Time Between Strokes*	x $\leq$ 0.314	0.363
	First Order Feature(s)		
	Time from Start to First Word Said	x $\leq$ 7.14	0.494
Stroop Word Color	Root Feature		
(Executive Function)	*Total Colors Generated*	x $\leq$ 6.5	0.363
	First Order Feature(s)		
	Average Time for Correct Response	x $\leq$ 1.921	0.124
	Average Time for Incorrect Response	x $\leq$ 19.569	0.444
Visuospatial	Root Feature		
(Executive Function)	*Total Distance Drawn*	x $\leq$ 3529.34	0.375
	First Order Feature(s)		
	Total Distance Drawn	x $\leq$ 2214.88	0.117
Visuospatial	Root Feature		
with Cognitive	*Average Distance from Correct Path*	x $\leq$ 13.332	0.375
Interference	First Order Feature(s)		
(Executive Function)	*Time*	x $\leq$ 17.493	0.278

Appendix Table 6. Features of Interest given Decision Tree Results for Multifunctional Tests - Gini Index Values of Functional Neurocognitive Assessment Tests for PD Population versus Controls.
